# A systematic review of global experiences with Medtronic’s Hugo™ robotic system for robot-assisted partial nephrectomy

**DOI:** 10.1007/s11701-025-02818-z

**Published:** 2025-10-02

**Authors:** Saif Toubasey, Mehrshad Sultani Tehrani, Andrew Shepherd, Noah Beetge, Arun Thakur, Mughilan Muralitharan, Mohammed Hegazy, Samuel Davies, Ben Challacombe

**Affiliations:** 1 King’s College London, London, UK; 2 Royal Adelaide Hospital, Adelaide, Australia; 3 Adelaide Medical School, University of Adelaide, Adelaide, Australia; 4 Guy’s and St Thomas’ NHS Foundation Trust, London, UK

**Keywords:** RAS, RAPN, Hugo, da Vinci, Robotic-assisted partial nephrectomy

## Abstract

Robotic-assisted partial nephrectomy (RAPN) has become a widely used modality for the excision of renal tumours. Medtronic’s Hugo™ Robotic-Assisted Surgery (RAS) system has emerged as a promising alternative to the established Intuitive da Vinci® platform. Due to increasing use, this systematic review has been conducted to assess the safety and efficacy of the Hugo™ RAS system for RAPN. A comprehensive search was conducted to identify eligible studies that reported outcomes and experience of using the Hugo™ system for RAPN. Following appropriate screening and risk of bias assessment using ROBINS-I, results were collated into a narrative synthesis. Eight studies were included in the review. Within these, 145 patients had undergone RAPN using the Hugo™ system. These studies comprised one comparative and seven single-arm case series. Patient demographics, peri-operative outcomes, pathological outcomes and safety and feasibility parameters were extracted from the included studies. Across all these metrics, the Hugo™ system performed well. One notable finding was longer docking times compared to the da Vinci® system; however, with increasing experience and familiarity with this novel platform, this is expected to decrease. Despite the lack of high-quality evidence and few studies, the Hugo™ RAS system is a feasible option for conducting RAPN. It offered comparable and promising initial outcomes in several aspects compared to da Vinci®, alongside flexibility in docking setup. Further large-scale comparative studies are needed to corroborate the findings of this review and evaluate the longer term safety and efficacy of the Hugo™ RAS system for RAPN.

## Introduction

Minimally invasive surgery has become the gold standard treatment option for many pathologies since its introduction in the 1980s, transforming patient care and offering several improvements such as shorter recovery times, reduced complication rates and improved peri-operative outcomes [1, 2]. This began as laparoscopic surgery; however, in more recent years, robotic surgery emerged as a groundbreaking new option in multiple specialties.

Urology was an early adopter of robotic technologies, and this continued innovation has seen the range of indications expanded to include robotic-assisted partial nephrectomy (RAPN) [3]. This procedure is increasingly being performed using robotic systems as they allow for improved precision, dexterity and control, allowing for the maximum preservation of normal kidney function whilst optimising oncological outcomes [4].

Historically, Intuitive Surgical’s da Vinci® robotic system has been the primary platform utilised for robotic surgery since it first received FDA approval in the year 2000 [5]. It has consistently been at the forefront of robotic surgery, setting a high benchmark, recently launching its newest iteration, the da Vinci® 5 [6]. The da Vinci® Surgical system had a market monopoly due to patents which only expired in 2019, ending its dominance [7]. This has allowed new systems to emerge and drive further innovation and competition in the robotic surgery sphere.

Medtronic’s Hugo™ Robotic-Assisted Surgery (RAS) system stands out as one of the competitors, with its first clinical use in June 2021 [8]. This surgical system has emerged as one of the most promising competitors to the da Vinci® for RAPN owing to the modularity of the arm carts, allowing for adaptations to various setup configurations for different surgical access needs [9]. Another factor which has contributed to the Hugo™ system’s popularity is its open console, providing easier communication with the rest of the surgical team.

Despite the Hugo™ platform’s growing popularity, there remains the critical question of its safety and efficacy generally and in comparison to other systems such as the da Vinci® [10].

Since first being described in 2004, RAPN has been increasing in popularity and becoming a more common approach rivalling open or laparoscopic approaches [11]. RAPN has been shown to have lower complication rates and reduced blood loss compared to open partial nephrectomy, as well as shorter warm ischaemic times than laparoscopic partial nephrectomy [12, 13]. With greater experience, more complex tumours can now be treated robotically, gaining the benefits of a minimally invasive approach, improving peri-operative outcomes and patient recovery, compared to open surgery.

Due to the novelty of the Hugo™ platform, there are few studies demonstrating its capabilities in performing RAPN. Although early in its clinical use, we believe that a systematic review is warranted that consolidates the existing evidence, bringing together the results from different studies to better assess the system’s safety and efficacy. It is our belief that this review will provide a clearer understanding to clinicians on how it may compare to other systems or surgical approaches and evaluating its outcomes to date.

## Methods

This systematic review adheres to the Preferred Reporting Items for Systematic Reviews and Meta-analyses (PRISMA) guidelines for review protocols [14]. The review was registered in Prospero (the international prospective register of systematic reviews); reference ID: CRD42025640573.

A comprehensive search strategy was used to find relevant articles for our study, to assess the efficacy and safety of the Hugo™ robotic system (see Appendix 1).

The population for our study was defined as adult patients (18 years or older) with renal cancer, who underwent RAPN. The intervention is Medtronic’s Hugo™ RAS system, whilst the comparator is existing robotic surgical outcome data using other robotic-assisted surgical systems, where possible.

After an initial scoping search, terms and the search strategy were further optimised to be comprehensive. The following databases were searched for studies: Medline, Embase, Scopus, Web of Science, Cochrane Central Register of Controlled Trials (CENTRAL), ClinicalTrials.gov and World Health Organization (WHO) International Clinical Trials Registry Platform (ICTRP). In addition to the studies found in these databases, manual forward and backward citation searching was conducted in studies identified to be eligible. All studies were then collected into Endnote Clarivate for analysis. Any duplicates uploaded to the system were identified and removed.

Once these steps had been completed, title and abstract screening were undertaken, followed by full-text screening. Screening of all identified studies was conducted by two independent authors (ST, MST), with any disagreements or discrepancies in findings resolved through discussion with a third reviewer (BC), where necessary.

In the review, we included case–control studies, cohort studies, case series, randomised control trials, other systematic reviews and meta-analyses that were relevant and met our inclusion criteria. We then went on to the full-text review of included studies, including those with direct comparisons between data from robotic platforms and descriptive comparisons between Hugo™ experiences and current practice. We decided not to exclude studies that were available as abstracts only. Our exclusion criteria included the following: studies not written in English or that do not have a full English translation, letters/commentaries/editorials, and conference abstracts without sufficient data. Our full-text review was carried out in accordance with the predetermined PICO criteria outlined in the review’s protocol [15]. 

Once the studies were selected, three reviewers (ST, MST, NB) conducted data extraction. The data extracted included study characteristics, participant information, interventions conducted and the outcomes of the procedures.

Risk of bias assessment was also carried out by three reviewers (ST, AT, MM) using the Robins-I V2 approach (Risk of Bias in non-randomised Studies—of interventions) [16]. This framework assessed 7 domains: confounding, classification of interventions, participant selection, deviation from intended interventions, missing data, outcome measurement and reported result selection. This model helped us assess the quality of the selected studies and to determine which aspects of the studies most contributed to bias.

## Results

**Fig. 1:**
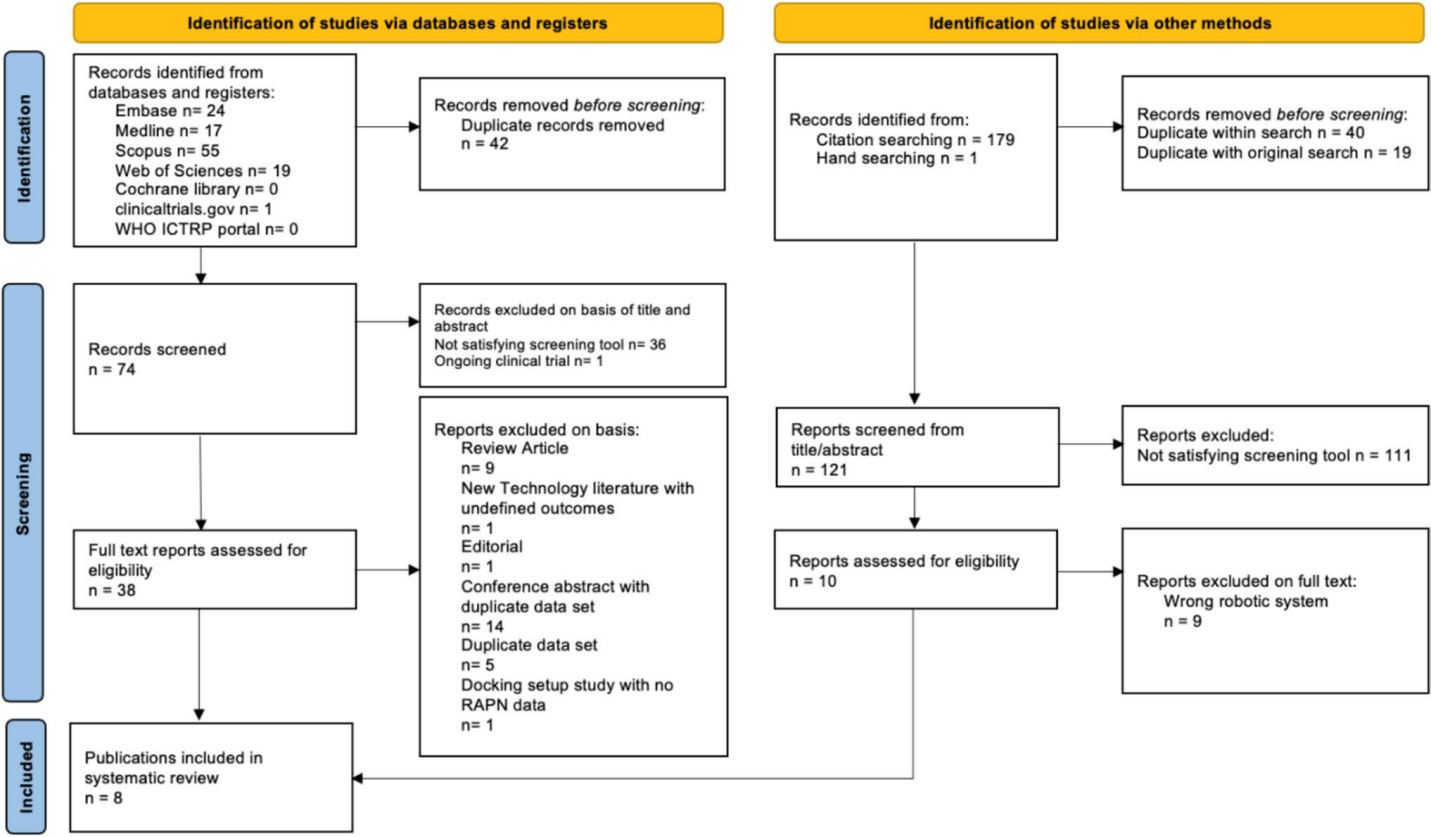
PRISMA flow diagram of literature search and systematic review process

Figure 1 shows our PRISMA flow diagram documenting how many studies we had in each stage of our screening. Our screening tool, after title and abstract screening, found 38 potentially relevant studies, with 7 deemed eligible for systematic review after conducting full-text review alongside an additional study found through hand searching, bringing the total to 8.

The eight studies that were included in this systematic review were published from 2023 to 2025 and an overview of each of them is presented in Table 1. From these included studies, one was a comparative study, comparing the Hugo™ to the da Vinci® (Rojo et al.) [17, 18]. One study was a singular case report (Tedesco et al.) [19]; however, it was included due to it presenting an interesting insight into an off-clamp partial nephrectomy using the Hugo™ for a challengingly large mass. The remaining focused on the feasibility, safety and peri-operative outcomes of using the Hugo™ for RAPN without directly comparing it to another system. These six studies included three prospective case series. Chierigo et al. [20] discussed both transperitoneal and retroperitoneal approaches, whilst Gallioli et al. [21] discussed initial experiences with the Hugo™ RAS. The third, an abstract by Shepherd et al., discussed outcomes of using the Hugo™ for upper tract urological procedures, including RAPN [22]. The remaining three studies were two retrospective case series by Prata et al. [23] & Bobrowski et al. [24] and a case report series by Gaya et al. [25], which all discussed the efficacy of the system as well as the flexibility of its setup.

**Table 1:** Characteristics of included studies

Author	Country	Robotic platforms	Study type	Sample size (Hugo™/da Vinci®)	Main aim	Outcome
Bobrowski, A. et al. 2024	Canada	Hugo™	Retrospective case series	11	Evaluate the feasibility, safety, and outcomes of robotic-assisted partial nephrectomy using the Hugo™ robotic-assisted surgery platform	Peri-operative parameters comparable to published literature with absence of intra-operative complications or conversions
Chierigo, F. et al. 2024	Italy	Hugo™	Prospective case series	10	Evaluate the feasibility, safety, and perioperative outcomes of transperitoneal and retroperitoneal robot-assisted partial nephrectomy using the Hugo™ robotic-assisted surgery platform	Versatility of Hugo™ allows for feasibility of transperitoneal or retroperitoneal approaches for RAPN, without compromising complication rates
Gallioli, A. et al. 2023	Spain	Hugo™	Prospective case series	10	Describe the surgical setting and assess the feasibility of robot-assisted partial nephrectomy using the Hugo™ robotic-assisted surgery platform	Variety of trocar placement setups for RAPN assessed are practical aside from one too lateral leading to arm clashes
Gaya, J. M. et al. 2023	Spain	Hugo™	Case report series	3	Describe the surgical setting and feasibility of retroperitoneal robot-assisted partial nephrectomy using the Hugo™ robotic-assisted surgery system	Determined optimal setting for trocars and arms in retroperitoneal RAPN with satisfactory peri-operative parameters
Prata, F. et al. 2025	Italy	Hugo™	Retrospective case series	80	To evaluate perioperative and early functional outcomes of RAPN using the HugoTM	System is safe and feasible for management of renal masses including complex cases, demonstrating preliminary favourable outcomes
Rojo, E. G. et al. 2024	Spain	Hugo™ & da Vinci®	Prospective comparative study	25 (Hugo™) / 25 (da Vinci®)	Compare perioperative outcomes of RAPN between Hugo™ and da Vinci® platforms	Comparable peri-operative, pathological, and functional outcomes present between the Hugo™ and da Vinci® platforms
Tedesco, F. et al. 2025	Italy	Hugo™	Case report	1	Demonstrate feasibility and safety of off-clamp robot-assisted partial nephrectomy for T2 renal mass using Hugo™	Safe and feasible alternative for excision of challenging large masses via RAPN
Shepherd, A et al. 2024	England	HugoTM	Prospective case series	5	Assess the safety of device without converting to another robotic platform, laparoscopy or open. Secondary outcomes assessed surgical and oncologic outcomes	Safe introduction of system for RAPN, good oncological outcomes

### Risk of bias

**Fig. 2:**
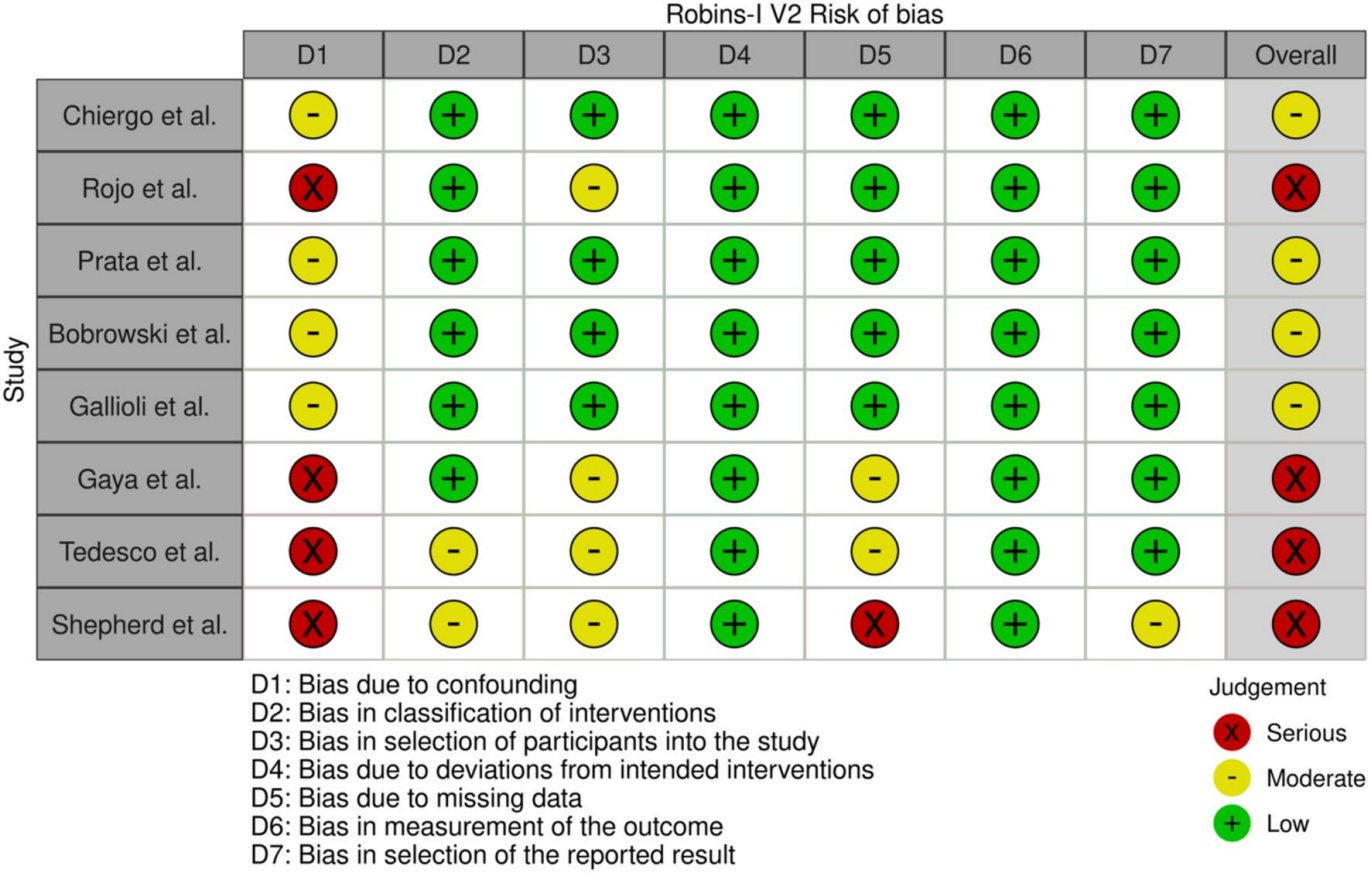
Robins-I V2 Risk of bias

**Fig. 3:**
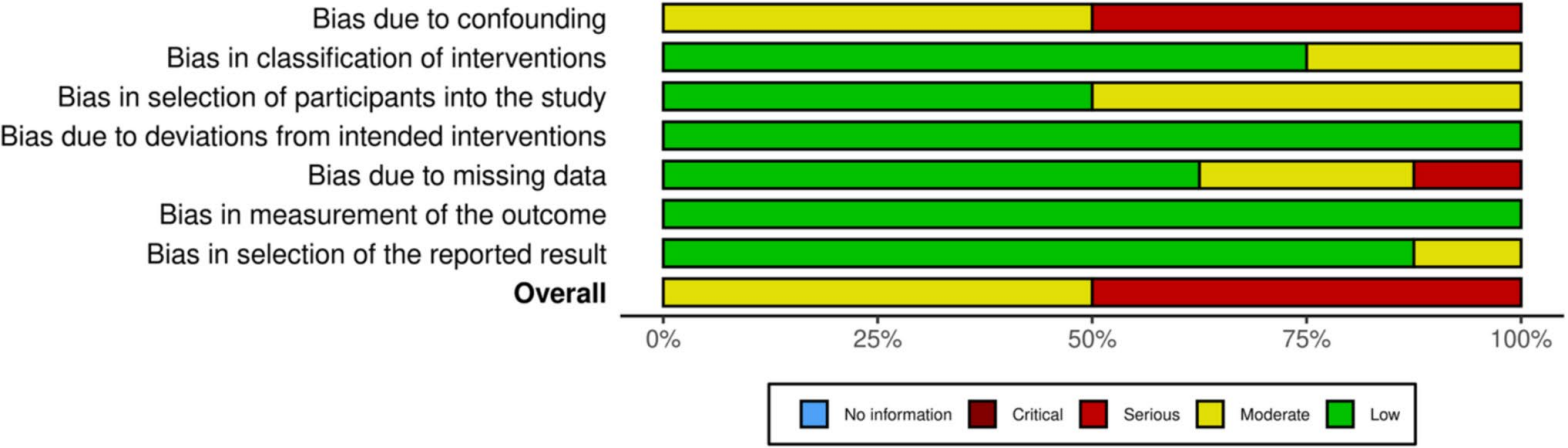
Risk of Bias summary plot

Risk of bias analysis, using the Robins-I V2 tool, was carried out to assess the quality of included studies. The tool showed that 4 studies were found to demonstrate a serious risk of bias and 4 at a moderate risk. The results of the Robins-I V2 risk of bias analysis are seen in Figs. 2 and 3 utilising the Robvis tool [26].

The main domain to affect bias was found to be confounding; this is a result of the studies lacking randomisation. Bias due to selection of participants in the study was also a factor for risk in a number of the studies due to some containing minimal cases which seemed to be hand-chosen to trial on the Hugo™ RAS system, understandable when considering safety implications for such a novel system. Moreover, bias due to missing data was another factor for moderate risk in 2 of the studies due to some data with regard to operative outcomes being missing, though reporting of composite binary outcomes such as ‘trifecta achievement rate’ was described in some. There was also a serious risk of bias for this category in one study, the Shepherd et al. study; however, this may be due to the fact it was a conference abstract, so could not include all the data ideally required [22].

### Operative timings

Total operative time ranged from 91 to 185 min. This seemingly large discrepancy once again arises from differing techniques and approaches, as well as some studies taking longer due to the included cases being some of the first RAPN the surgeons had conducted with the Hugo™ or were trialling different arm cart configurations. In the study by Rojo et al., the console time between the Hugo™ (103.15 min) was very similar (*P* < 0.86) to that of da Vinci® (102.28 min). This shows that the Hugo™ was a very adept alternative to the mainstay system for RAPN. The Prata et al. study contained a subgroup within the cohort of 36 moderate-to-high complexity, which had a median operative time of 135 min.

Table 2 includes the selected studies which reported operative and docking times. These Hugo™ studies concluded that it is an efficient and adequate system for conducting RAPN and may become more effective as surgeons adapt to the console.

**Table 2:** Summary of timings in included studies

Study	Operative time Hugo™, minutes (median, IQR)	Operative time other, minutes (median IQR)	Docking time minutes (mean, SD)	Findings
Bobrowski, A. et al	Total 165.5 (34.1) [Mean, SD] Console 93 (21.4) [Mean, SD]	N/A	232secs (106.5)	This relatively low time reflects the efficiency of the Hugo™ platform in performing RAPN in their small cohort of 11 patients
Chierigo, F. et al	Total: 185 (170,232) Console: 136 (100,159)	N/A	20 (15–23) [Median, IQR]	The study suggested that multiple arm carts lead to longer setup times (docking). The Hugo™ system provides a reasonable operative time for RAPN
Gallioli, A. et al	Console: 138 (124–162)	N/A	9.5 (9–14) [Median, IQR]	Hugo™ system provides a reasonable operative time for RAPN, with no intraoperative complications reported
Gaya, J. M. et al	Console: 180 (160–210)	N/A	6 (6–7) [Median, IQR]	Versatility for both transperitoneal and retroperitoneal approaches
Prata, F. et al	Total: 105.5 (78–149)	N/A	4(3–6) [Median, IQR]	Operative timings align with da Vinci® benchmark
Rojo, E. G. et al	Console: 103.15 (46.51) Hugo™	Console: 102.28 (45.07) da Vinci® [Mean, SD]	20.08 (6.31) Hugo™; 12.56 (2.98) da Vinci®	Docking time was significantly shorter in the da Vinci® group (P < 0.01), while console time was similar between both (P < 0.86)
Tedesco, F. et al	Console: 96	N/A	3	A singular case study, showed system can efficiently carry out off-clamp RAPN

*SD* standard deviation, *IQR* interquartile range

### Docking timings

The Hugo™ system differs from the da Vinci® system in that its setup involves arranging the modular arm carts separately; this allows for greater versatility at the potential expense of a prolonged docking time when initially learning the platform. In the included studies, the docking time for the Hugo™ ranged from 3 to 20 min. This shows that there is a certain degree of variability in this aspect of using the robot, most likely due to surgeons having varying levels of experience and exposure to the system. In the study by Rojo et al. comparing the two platforms, docking time for da Vinci® (12.56 min) was statistically significantly lower (*p* < 0.01) than that of Hugo™ (20.08 min); this is most likely as a result of the surgical team being more familiar with the da Vinci®.

### Patient demographics

The average age of the patients included in the studies ranged from 58 to 68, excluding the patient from Tedesco et al. that was an outlier aged 25 [19]. This is attributable to it being a case study of a difficult off-clamp case. All the studies which stated gender split had a higher proportion of male patients than female patients, which is to be expected due to the higher incidence of renal cancer in males [27].

The studies did not use any specific criteria to select cases for the Hugo™ system but rather would decide if a case could be conducted using minimally invasive techniques. The only exception being the Chierigo et al. study which claimed that complex cases were chosen to be conducted using the da Vinci® while familiarising with the Hugo™ system. The study by Rojo et al. stated that the allocation of patients to Hugo™ or da Vinci® was done completely logistically based on robotic availability, rather than patient or surgeon’s preference. This is important as it makes the studies more generalisable and allows for higher quality comparisons.

The average CT tumour size for the studies (excluding the singular case study) ranged from 2.75 cm to 3.5 cm. The average RENAL score of patients included in the study ranged from 5 to 7. These two sets of data show that the majority of patients included in the study had relatively small, exophytic tumours which were considered of low-to-moderate complexity.

BMI was also recorded in some of these studies, including the comparative study. Rojo et al. found that their Hugo™ cohort had a higher average BMI (27.9) in comparison to the da Vinci® group (26.5). Although this difference was not statistically significant and was not purposefully done, it does raise the point that the Hugo™ RAS could be favourable in patients with a larger body habitus, due to being able to move and adapt the positioning of all the separate arms. All the patient demographic information collated from the studies can be seen in Table 3.

**Table 3:** Summary of patient demographics and tumour characteristics in studies

Study	Age (median, IQR)	Gender	Ct tumour size cm (median, IQR)	ASA score	R.E.N.A.L. score (median, IQR)	PADUA score (median, IQR)	BMI (median, IQR)
Bobrowski, A. et al	58 (18)	F5, M6	2.9 [Mean]	3	5 (4,8)	N/A	28.3 (13.7)
Chierigo, F. et al	58 (54,66)	F2, M8	2.75	2 (2,2)	6 (5,8)	7 (7,9)	24.00 (21.75, 25.75)
Gallioli, A. et al	68 (61–75)	F6, M4	3.0 (2.2–3.7)	N/A	N/A	9 (8,9)	N/A
Gaya, J. M. et al	N/A	N/A	3.2 (2.7–5.6)	N/A	N/A	7 (7,8)	N/A
Prata, F. et al	63 (55–72)	F27 M53	3.25 (2.5–5)	2, (2,3)	6 (5–8)	N/A	27 (24.3–31.1)
Rojo, E. G. et al	62.52 (9.47) [Mean]	F15, M35	3.5 (2.13) Hugo™, 3.29 (1.166) da Vinci® [Mean, SD]	2(1,3)	5.76 (2.81) Hugo™, 5.60 (2.12) da Vinci®	6.88 (3.01) Hugo™, 7(2.08) da Vinci® [Mean, SD]	27.9 (3.89) Hugo, 26.5 (4.11) da Vinci® [Mean, SD]
Shepherd, A et al	N/A	N/A	N/A	N/A	N/A	8	N/A
Tedesco, F. et al	25	M1	9.5 [mean]	N/A	N/A	11	N/A

### Safety and feasibility

As with any new medical device or treatment, the primary concern is with safety and efficacy of the novel method, particularly in comparison to the existing gold standard. To assess the safety and feasibility of the Hugo™ RAS, peri-operative and post-operative data was extracted from the studies included in this review, such as blood loss, positive surgical margins, warm ischaemic times and complications, as well as any conversions to laparoscopic or open surgery. These data are summarised in Table 4.

**Table 4:** Summary of operative outcomes

Study	EBL mL (median, IQR)	WIT minutes (median IQR)	PSM, n (%)	Intra-op complication/conversions	Post-operative complications
Bobrowski, A. et al	179 (63.6) [Mean, SD]	18.9 (7.12) [Mean, SD]	0	N/A	1 wound infection CD grade II
Chierigo, F. et al	100 (50,138)	16 (15,20)	0	N/A	CD: 1 × Grade I, 1 × Grade II, 2 × Grade IIIa
Gallioli, A. et al	90 (75–100)	13 (10–14)	0	1 × conversion to laparoscopic (suboptimal trocar placement + hepatomegaly). Right RAPN of a 6 cm renal mass	CD 3a (bleeding due to pseudoaneurysm) 2 × CD grade II
Prata, F. et al	200 (100–0)	OFF CLAMP	3 (3.8%),	12 (15%)	4 × CD I (fever), 7 × CD II (transfusions), 1 × CD IIIa (pseudoaneurysm)
Rojo, E. G. et al	142.4 (85.25) Hugo™, 233.8 (265.75) da Vinci® [Mean, SD]	9.92 (11.94) Hugo™, 14.38 (15.61) da Vinci® [Mean, SD]	Hugo 8%, da Vinci® 4%	N/A	1 × CD I–II in da Vinci® and 1 × CD III-IV in Hugo and da Vinci®
Tedesco, F. et al	350	OFF CLAMP	0	N/A	No complications
Shepherd, A et al	N/A	19 (17,20)	0	Temporary non-functional arm due to accidental activation of mechanical release	No complications

*CD* Clavien–Dindo

The average estimated blood loss for Hugo™ cases from the studies included ranged from 75 mL to 179 mL (excluding the Tedesco et al. study which included an off-clamp RAPN case). In the Rojo et al. study the blood loss for da Vinci® was noticeably higher than that of the Hugo™, however, was not statistically significant (*p* = 0.41). These findings can lead us to believe that regarding estimated blood loss, the Hugo™ RAS performs in line with other current methods of carrying out partial nephrectomies, making it a viable option.

The study by Gallioli et al. was the one in our review to have any cases of conversion from Hugo™ RAS. One case of a partial nephrectomy conversion was reported, where the procedure became laparoscopic due to suboptimal trocar placement, leading to continuous collisions, as well as due to the patient’s hepatomegaly [21]. This case shows the importance of correctly positioning the robotic arm carts and ensuring trocar placement is correct. This patient also developed bleeding due to a pseudoaneurysm on day 3 post-op. Shepherd et al. also highlighted a temporary non-functional arm, however, this was due to accidental activation of the mechanical release for the instrument drive unit and did not lead to any intra-operative complications [22]. There were no other intraoperative device failures reported or any other cases the required a conversion laparoscopic or open surgery.

Throughout the studies, there were some complications, with grading varying from Clavien–Dindo grade I–IIIa (e.g. wound infection). These complications cannot be entirely attributable to the new platform as they are recognised risks of the surgery itself.

Regarding positive surgical margins, only two studies noted any, Rojo et al. (2 cases) and Prata et al. (3 cases). Out of 145 RAPN cases conducting using the Hugo™ only 5 patients had positive surgical margins (3.4% of cases) which is in line with or below normal estimates for partial nephrectomies [28].

## Discussion

The limited current literature describing experience with the new Hugo™ RAS platform for RAPN demonstrate feasibility and indicate safety. The included studies demonstrate comparable perioperative outcomes, operative and console times to the da Vinci® as well as minimal serious post-operative complications. This is demonstrated by the fact that only 3.4% of cases included within the review were found to have positive surgical margins which is below the PSM rate noted (4.3%) in previous large-scale RAPN studies using alternative systems [29]. A systematic review conducted comparing outcomes of RARP cases using both the Hugo™ and da Vinci® also similarly found no statistically significant differences between the two consoles [30]. Despite being for a different procedure, the study does align with our findings that the Hugo™ can produce similar PSM rates to da Vinci®.

The study by Rojo et al. did demonstrate that docking times were longer for the Hugo™ RAS compared to the da Vinci®, which is less of a drawback when considering the novelty of the device, such that as experience grows, docking durations would be expected to reduce (as seen in the Hugo RAS robotic-assisted radical prostatectomy literature) [31, 32]. This longer docking time is due to the docking process being more challenging as a result of the arms being on individual carts, so must be placed in the correct position independently [33]. Also as shown in a study investigating docking times for the Hugo™, as experience grows with the system, ‘docking times showed a significant negative trend (*p* < 0.01)’ [34]. This shows that potentially as the Hugo™ system’s use becomes more widespread and standardised, docking times could become more comparable with da Vinci®. Despite a significantly longer docking time, the Ditonno et al. systematic review for RARP cases conducted using the Hugo™ found that this did not have a statistically significant difference or impact on overall operative times [30].

**Fig. 4:**
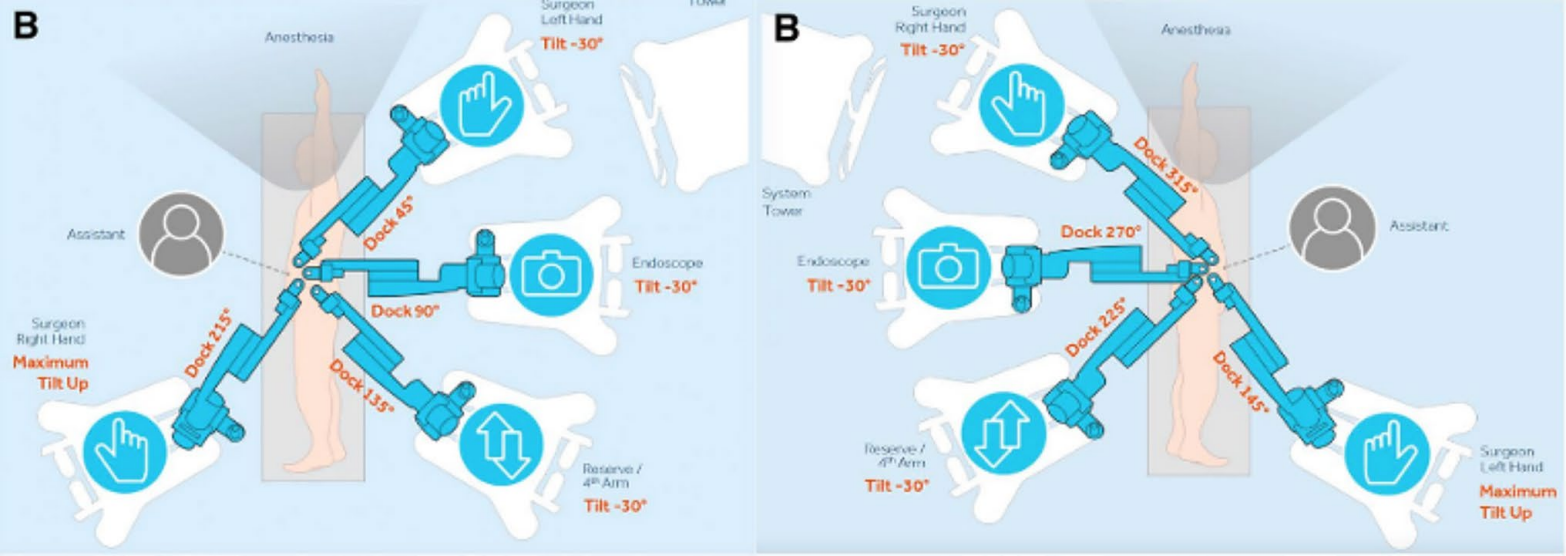
Trocar placement for left RAPN (left) and right RAPN (right) from Gallioli et al. [21]

Another note that is highlighted throughout the studies is the flexibility that the Hugo™ system offers with the 4 modular arm carts. Each arm can be adjusted separately depending on the patient’s anatomy and body type, proving to be a useful tool in patients with a larger body habitus [35, 36]. This can be visualised in Fig. 4. Also, the study by Gaya et al. noted that this increased the system’s versatility, making it feasible to be used for both transperitoneal and retroperitoneal approaches. There is limited literature currently discussing whether different arm configurations could lead to differences in surgical time, but this could be a scope for future research as the system becomes adopted more widely.

Although there have been concerns discussed about reduced working space for the bedside assistant, the Bobrowski et al. study highlighted that raising the patient bed could help mitigate the issue. The Hugo™ system has longer arms, so raising the patient bed could allow the assistant to operate underneath the movement of the arms, reducing the risk of collisions. The Prata et al. paper also notes that the modular arm cart nature necessitated closer coordination with the bedside assistant; however, after the first 15 cases, a reliable and streamlined workflow was achieved.

A few of the studies within this systematic review mentioned the improved ergonomics brought about by the Hugo™ system. Both the Rojo et al. and Gallioli et al. studies mentioned how renorrhaphy was easier to conduct using the Hugo™, due to the double rotational capacity (up to 529 degrees), allowing faster and more comfortable suturing. Safety features such as collision prevention and force-sensing technologies were also noted as appreciated features of the system. The open console design was also mentioned throughout the studies as a positive for the Hugo™ system, giving the surgeon a greater awareness of his surroundings, as well as improving communications with the rest of the team. In the future, further research into specific ergonomics, such as surgeon comfort and posture while conducting RAPN procedures, would be beneficial.

A potential negative change that the Hugo™ has compared to da Vinci® is regarding latency. There have been no reported issues in relation to motion transmission or feedback. However, Bobrowski et al. and Chierigo et al. both discussed a slight delay when activating monopolar or bipolar diathermy compared to the da Vinci®. This has also been noted by our centre’s experience using the Hugo™, as well as being discussed at the recent ERUS25 conference, where a safety feature leads to the energy taking a moment to activate after being pressed. Therefore, this is an area that requires an adjustment period for surgeons transitioning from da Vinci®.

Our review highlights the need for more high-quality studies of Hugo™ RAS for RAPN. No high-quality randomised trials have yet been published. This demonstrates a gap in the literature to more fully assess the efficacy and feasibility of the system for RAPN and renal surgery, more broadly. There is currently one ongoing randomised trial comparing outcomes of da Vinci® and Hugo™ for renal surgery; therefore, progress is already being made in this regard [37]. The lack of studies was further highlighted by the risk of bias analysis, which showed the majority of studies included had a moderate-to-high risk of bias, demonstrating a potential limitation of our study. This risk of bias indicates that patient cohorts may be highly selected (which is often the case when introducing a new system), potentially underestimating complication rates and inflating apparent feasibility. However, once again, this is only due to the novelty of the platform and as more centres take it up and more cases are conducted with the Hugo™ system, we expect this to improve.

When extracting the quantitative data from the literature included in this study, a high degree of heterogeneity was found, for example, the reporting of warm ischaemic times, whilst others used trifecta outcome percentages instead. The limited number of studies, alongside the high degree of heterogeneity, made direct comparisons more difficult and prevented us from conducting a meta-analysis.

Another point this review highlights is the fact that most of the current literature surrounding the topic comes from centres with significant experience conducting partial nephrectomy procedures using the da Vinci® system. The studies included are using data from high-volume robotic centres and show that prior experience using a different robotic platform can transition to the Hugo™ system without many difficulties and with a short learning curve, with other studies into other renal procedures also demonstrating a straightforward skills transfer between the two systems [38]. This, however, limits the generalisability of the study’s findings and results, as the experiences seen in these high-volume robotic centres may not reflect a robotic-naïve centre’s initial experiences using the Hugo™ RAS system. Therefore, what is indicated by this review is the need for further data focusing on outcomes of partial nephrectomy procedures carried out by novice and new robotic surgeons or centres to see how these compare to the findings from the studies included within this review.

Despite the lack of high-quality evidence to date for Hugo™ RAS, there appears to be an increasing interest in the system, evidenced by a growing number of system installations worldwide, being used in Chile, Australia and New Zealand, Europe, as well as now several parts of Asia [39–41].

For future research and assessment of outcomes of RAPN procedures using the Hugo™ RAS, it would be valuable to have a standardised method of outcome reporting, such as using the trifecta, seen in the Prata et al. study, or Pentafecta scoring system, to make it easier to compare results between studies, as well as with other systems. Cost evaluation of the system is another area that should be explored more heavily in the future; in our review, only the Chierigo et al. study mentions a lower cost of €2700 per procedure using the Hugo™ compared to the da Vinci® procedure cost of €4700. This is only true of that specific centre therefore wider studies should be conducted to explore this important factor. Moreover, as longer term studies are conducted, it would be of great value for these studies to report long-term follow-up to further our understanding of the feasibility of the system and long-term oncological outcomes.

## Conclusions

We aimed to assess the efficacy, feasibility and safety of Medtronic’s Hugo™ RAS system RAPN, looking at peri- and post-operative outcomes and where possible, comparing to the da Vinci® platform. Several parameters including operative time, blood loss and complications were examined. The findings of this review show that the system is safe and feasible for use in RAPN. However, due to the relatively limited data and evidence, this review underscores the need for large, randomised controlled trials to fully evaluate the effectiveness of the Hugo™ system in comparison to other platforms for RAPN. Such studies will be crucial for establishing long-term efficacy, cost-effectiveness, and optimal patient selection criteria. To add to this, it would also be of great benefit if future studies could evaluate ergonomics, team dynamics and system latency in more depth, comparing how the Hugo™ system compares to other systems in this metric, as these are essential factors influencing whether the system will have widespread adoption.

To conclude, the findings of this review encourage wider adoption of the Hugo™ RAS system for RAPN, demonstrating its safety and feasibility in early series, whilst also highlighting the need for larger-scale studies to further evolve and refine the platform.

## Data Availability

No datasets were generated or analysed during the current study.
